# Biplex quantitative PCR to detect transcriptionally active human papillomavirus 16 from patient saliva

**DOI:** 10.1186/s12885-024-12125-9

**Published:** 2024-04-10

**Authors:** Fiona Deutsch, Dayna Sais, Ni Keatinge, Meredith Hill, Ngoc Ha Tran, Michael Elliott, Nham Tran

**Affiliations:** 1 School of Biomedical Engineering, Faculty of Engineering and IT, University of Technology Sydney, Ultimo, Australia; 2 Chris O’Brien Lifehouse, Sydney, NSW Australia; 3 Sydney Medical School, University of Sydney, Sydney, NSW Australia

**Keywords:** HPV16, Oropharyngeal cancers, Saliva, Biplex Quantitative PCR, Viral diagnosis

## Abstract

Head and neck cancers, particularly oropharyngeal cancers (OPC), have been increasingly associated with human papillomavirus (HPV) infections, specifically HPV16. The current methods for HPV16 detection primarily rely on p16 staining or PCR techniques. However, it is important to note the limitations of conventional PCR, as the presence of viral DNA does not always indicate an ongoing viral infection. Moreover, these tests heavily rely on the availability of tissue samples, which can present challenges in certain situations. In this study, we developed a RT-qPCR biplex approach to detect HPV16 oncogenes E6 and E7 RNA in saliva samples from OPC patients. Salivary supernatant was used as the liquid biopsy source. We successfully obtained RNA from salivary supernatant, preserving its integrity as indicated by the detection of several housekeeping genes. Our biplex approach accurately detected E6 and E7 RNA in HPV16-positive cell lines, tissues, and finally in OPC salivary samples. Importantly, the assay specifically targeted HPV16 and not HPV18. This biplexing technique allowed for reduced sample input without compromising specificity. In summary, our approach demonstrates the potential to detect viable HPV16 in saliva from OPC patients. Since the assay measures HPV16 RNA, it provides insights into the transcriptional activity of the virus. This could guide clinical decision-making and treatment planning for individuals with HPV-related OPC.

## Introduction

Head and neck (HNC) constitute a substantial portion of the global cancer burden, with more than 890,000 new cases and approximately 450,000 deaths reported in 2018, ranking them among the top 10 most common cancers worldwide [1]. These malignancies encompass a variety of tumours located in the oral cavity, pharynx, and larynx, each with distinct risk factors and epidemiological patterns. Despite advancements in diagnosis and treatment, the overall five-year survival rate remains around 50% [2], highlighting the need for improved detection methods and therapeutic strategies.

Tobacco and alcohol consumption are widely recognised as the primary risk factors for HNC [3], with the synergistic effect of tobacco and alcohol consumption on HNC extensively documented. However, human papillomavirus (HPV) infection has been implicated as a key determinant in the development of oropharyngeal cancers (OPC) [4]. It is estimated that nearly 70% of OPC cases are HPV-positive, with a substantial proportion of these cases occurring in the tonsils [5]. Approximately 85% of HPV-positive OPC cases are infected with oncolytic variants, such as HPV 16 or HPV 18 [6]. These HPV-positive OPCs have been found to have distinct clinical characteristics when compared to HPV-negative tumours, particularly with respect to treatment response and overall survival rates [7, 8].

The incidence rates of HPV-related OPCs are rising in both developed and developing countries. In some regions, the incidence of HPV-positive OPC has now surpassed that of HPV-negative OPC cases [5]. Studies conducted the United States and Europe have demonstrated a sharp rise in the incidence of HPV-positive OPC, particularly in young adults. In the United States, the incidence of HPV-positive OPC has increased by 225% among young men in the last two decades [9]. In Europe, a similar trend has been observed, with incidence rates of HPV-positive OPC increasing by approximately 60% among young men in the last 10 years [10].

In developing countries, the incidence of HPV-related OPC is rising and is expected to continue to increase [11]. In low- and middle-income countries within both Asia and Africa, the incidence of HPV-positive OPC has been increasing at a faster rate than in developed countries, with HPV 16 and 18 being the most common high-risk types identified [11].

High-risk types of human papillomaviruses, including HPV 16 and HPV 18, code for several oncogenes, in particular, E6 and E7. Under normal conditions, E6 and E7 are expressed at low levels and are thought to function by creating conditions in the infected oral keratinocytes that favour replication of the virus [12]. At higher levels, these two oncoproteins have major effects on a variety of cellular functions that may lead to uncontrolled growth [13]. E6 is best known for its ability to bind to and mediate the degradation of the tumour suppressor p53 [14] and other targets involved in cellular apoptotic pathways [15]. As a consequence of these interactions, cells expressing E6 are much less likely to undergo apoptosis. E7 is known for its ability to bind to and inactivate the tumour suppressor Rb protein, disrupting its ability to regulate E2F transcription factors, resulting in disrupted cell cycle regulation [15].

The detection of HPV is a critical component in the diagnosis and management of HPV-related OPC. There are several techniques available for HPV detection, including polymerase chain reaction (PCR)-based methods; hybrid capture (HC) assays; in situ hybridisation (ISH); and p16INK4a (p16) detection using immunohistochemistry (IHC) [16, 17]. p16 detection is the most used method in the diagnosis of HPV-related OPC [18]. While p16 overexpression is a marker of HPV-associated malignancy, the interpretation of p16 results can be subjective and can be affected by inter-observer variability [19]. In addition, using the p16 detection method may produce false positive results, as p16 overexpression can occur due to other causes besides HPV infection [20]. This underscores the complexity of using p16 as a biomarker, where its overexpression is not solely indicative of HPV involvement. Moreover, p16 overexpression is a late event in HPV-associated carcinogenesis, meaning that, it may not be present in early-stage cancers [21].

PCR methods have been widely used for the detection of HPV16 [22–28]. However, the major problem with the PCR approach is that the detection of viral DNA does not indicate an active infection. The virus may be dormant, and patients, even though they test positive for HPV, may not go on to develop cancer [28]. Despite the various methods available for HPV detection, there is a lack of RNA-based HPV testing, which can indicate an active infection. Furthermore, most of these tests require a tissue biopsy, which may limit the scope of testing.

There has been a growing interest in using saliva as a liquid biopsy for diagnosing certain diseases [29–32]. Saliva is an ideal choice as it contains genomic material and a diverse population of biological particles, making it a “mirror to the body” that reflects both local and systemic conditions [33]. This makes it plausible that saliva may contain RNA released by head and neck cancer cells or HPV16 within the oral cavity.

To this end, our study aimed to develop and evaluate a probe-based biplex reverse transcriptase quantitative PCR (RT-qPCR) technique to identify viable HPV16 RNA in the saliva of patients with OPC. Detecting active virus in the saliva of OPC patients would be a valuable clinical tool that could aid in directing appropriate treatment strategies for these individuals.

## Materials and methods

### Cell lines

The cell lines used in this study included: squamous cell carcinoma from the cervix, SiHa (HPV16 positive); epidermoid carcinoma from the cervix, CaSki (HPV16 positive); adenocarcinoma of the cervix, HeLa (HPV18 positive); and ductal carcinoma of the mammary gland, MCF-7 (HPV negative). All cells were maintained in Dulbecco’s Modified Eagle Medium (DMEM) GlutaMAX™ (Thermo Fisher Scientific) with 1% glutamine, 10% fetal calf serum (Thermo Fisher Scientific*)* in a 37 °C incubator with humidified 5% CO2. Cells were passaged upon reaching approximately 80% confluency, typically every 2–3 days, to ensure optimal growth and viability. For passaging, cells were detached using 0.25% trypsin-EDTA solution (Thermo Fisher Scientific) and subsequently seeded at appropriate densities for continued culture or experimental use. All cell lines were regularly monitored for morphological consistency and tested periodically to confirm the absence of mycoplasma contamination.

### Tissue specimens

Tissue specimens were retrieved from patients treated for Squamous Cell Carcinoma (SCC) of the Oropharynx at Royal Prince Alfred Hospital, Sydney, between 2002 and 2006. The study was approved by the Research Ethics Committee at Royal Prince Alfred Hospital, Sydney, Australia (Protocol number X05–0270). Informed consent was obtained for the collection of fresh tissues. Immediately after surgical resection, tissues were snap frozen on dry ice and stored at − 70 °C. The histology of tissues was assessed by hospital pathologists. For this study, six fresh-frozen tissues samples were selected from p16 positive (*n* = 4) and p16 negative (*n* = 2) OPC specimens.

### Saliva specimens

All HNC saliva samples were obtained from patients through informed written consent with approval by the ethics board at Royal Prince Alfred Hospital, Sydney, between 2018 and 2022 (Ethics: X19–0195 and 2019/ETH11588). From each patient, 2 mL of unstimulated saliva was collected directly into sterile collection tubes (non-commercial kit) or a commercial kit (DNA/RNA Shield SafeCollect Saliva Collection Kit, Zymo Research). For this proof-of-principle study, we collected saliva specimens from p16 positive (*n* = 3) and p16 negative (*n* = 5) OPC patients.

### RNA isolation from cell lines

5X10^6^ cultured cells were homogenised by adding 1 mL of RNAzol® RT (Molecular Research Center). The homogenate was incubated for 5 minutes at 4 °C after the addition of 0.4 mL RNase-free water (Invitrogen, Thermo Fisher Scientific) for DNA, protein and polysaccharide precipitation, and centrifuged at 12, 000 x *g* for 10 minutes at 4 °C. The supernatant was then transferred to a fresh tube and 5 μL 4-bromoanisole (Molecular Research Center) was added for RNA purification. The sample was incubated for 3 minutes at 4 °C and centrifuged at 12, 000 x *g* for 10 minutes at 4 °C. RNA was precipitated by adding one volume of isopropanol (Sigma Aldrich) to the supernatant. The sample was incubated overnight at − 20 °C and then centrifuged at 12, 000 x *g* for 10 minutes. The supernatant was discarded, and the RNA pellet was washed twice with 75% ethanol (Sigma Aldrich) by centrifugation at 12,000 x *g* for 5 minutes. Lastly, the RNA pellet was solubilised in 20 μL of RNase-free water.

### RNA isolation from tissue

100 mg of fresh frozen tissue was diced with a surgical blade, homogenised with a mortar and pestle, and rinsed with 1 mL of RNAzol® RT. 0.4 mL water was added to the sample and centrifuged at 12,000 x *g* for 10 minutes to precipitate the DNA and proteins. The sample was purified using BAN and centrifuged again at 12,000 x *g* for 10 minutes. RNA was precipitated using isopropanol according to the above protocol, and the sample was incubated at − 20 °C overnight. The RNA was then washed with 75% ethanol twice, and the RNA pellet was resuspended in 20 μL of RNase-free water.

### RNA isolation from saliva

We adapted our previously published protocol for serum RNA isolation [34] to extract total RNA from saliva samples. After retrieving the saliva samples from storage at − 30 °C, samples were centrifuged at 1600 x *g* for 15 minutes at 4 °C. This process was done to separate the cellular debris. The salivary supernatant (400 μL aliquots) was homogenised with 1.5 mL Tri-Reagent RT-Liquid Samples (Molecular Research Centre) and 100 μL 4-bromoanisole, then centrifuged at 12,000 x *g* for 20 minutes at 4 °C in 1.5 mL phase-lock gel tubes (5PRIME).

The RNA-containing aqueous phase was decanted into a fresh DNA Eppendorf Lo-bind tube, mixed 500 μL isopropanol and 5 μL Glycogen, and incubated overnight at − 20 °C. Following incubation, samples were centrifuged at 12,000 x *g* for 20 minutes at 4 °C. The supernatant was discarded, and the RNA pellet was washed twice with 1 mL of 70% ethanol, air dried, and resuspended in 20 μL of RNase-free water. For increased yield, samples from the same participant were pooled.

### RNA quantification and quality control

Total RNA was quantitated using a Nanodrop™ 1000 3.7.1 UV-Vis Spectrophotometer (Thermo Fisher Scientific). After cleaning the stage with water and 70% ethanol, the instrument was blanked using 1 μL of RNase-free water. Using 1 μL of sample, the absorbance spectra were measured. RNA concentration was determined from the 260 nm peak, and purity was assessed using absorbance ratios at 280 nm (A_260_/ A_280_) and 230 nm (A_260_/A_230_). Accepted ratios for purity vary with downstream applications, however, typical A_260_/ A_280_ ratios should be between 1.8–2.2, while requirements for A_260_/A_230_ ratios are generally greater than 1.7.

### cDNA synthesis

The High-Capacity cDNA Reverse Transcription Kit from Thermo Fisher Scientific was used for cDNA synthesis. cDNA synthesis was performed using a 20 μL reaction, per Table 1, and employed a range of RNA input concentrations from 50 pg to 200 ng. Tubes were then placed in a thermocycler and run using the following conditions: 10 minutes at 25 °C, 120 minutes at 37 °C, 5 minutes at 85 °C and the sample was held at 4 °C until collected.

**Table 1:** cDNA synthesis components per 20 μL reaction

Component	Volume – 1x reaction (μL)
10x Reverse Transcriptase Buffer 1.0 mL	2.0
25x dNTP Mix 100 mM, 200 μL	0.8
RNase Inhibitor 100 μL, 20 Units/μL	1.0
RNA Input (various concentrations)	1.0
10x RT Random Primer, 1.0ML	2.0
MultiScribe™ Reverse Transcriptase 100 μL, 50 units/μL	1.0
Nuclease-free water	12.2
**Total**	20.0 μL

### Reverse transcriptase quantitative polymerase chain reaction (RT-qPCR); Biplexing HPV16 oncogenes E6/E7

Following cDNA synthesis, samples were diluted 1:4 by adding 60 μL nuclease-free water. RT-qPCR was then performed in a 5 μL reaction volume, per Table 2 using the StepOnePlus™ Real-Time PCR system (Thermo Fisher Scientific, USA). Reactions we performed in triplicate. The reactions utilised the TaqMan Universal PCR Master Mix (Applied Biosystems, Thermo Fischer Scientific, USA), adhering to the cycling conditions outlined in Table 2. TaqMan assays were designed for the oncogenes *E6* and *E7*, using Primer3Plus (https://primer3plus.com/) based on sequences obtained from NCBI (https://www.ncbi.nlm.nih.gov/refseq/). These sequences are outlined in Tables 3 and 4 below. To biplex these two oncogenes, E6 was labelled with a VIC™ and E7 was labelled with a FAM™ probe.

**Table 2:** RT-qPCR components per 5 μL reaction

Component	Volume – 1x reaction (μL)
TaqMan Universal PCR Master Mix (20X)	2.5
TaqMan Assay for E6/E7 (20X)	0.5
cDNA	1.0
Water	1.0
**Total**	5.0

**Table 3:** Primer and probe sequences E6

Component	Sequence
Context sequence	TGGACAAGCAGAACCGGACAGAGCC
Probe (VIC)	TCCGGTTCTGCTTGTCC
Forward sequence	GCTCAGAGGAGGAGGATGAAATAGA
Reverse sequence	GAGTCACACTTGCAACAAAAGGTT

**Table 4:** Primer and probe sequences E7

Component	Sequence
Context sequence	ACCCAGAAAGTTACCACAGTTATGC
Probe (FAM)	ACAGAGCTGCAAACAA
Forward sequence	ACCCAGAAAGTTACCACAGTTATGC
Reverse sequence	TGCTTGCAGTACACACATTCTAAT

A 2-step PCR assay was employed for its enhanced specificity and flexibility, particularly beneficial for biplexing the oncogenes E6 and E7. This approach allowed for separate optimisation of reverse transcription and PCR amplification conditions, improving assay sensitivity, and reducing potential interference from RT reaction components during the PCR step. To ensure assay accuracy and prevent contamination, each RT-qPCR run included both positive and negative controls. Positive controls comprised known quantities of target cDNA (Siha and CaSki) to verify PCR efficacy, while negative controls (no-template controls) contained all reaction components except the template cDNA, serving to detect any potential contamination or non-specific amplification. These controls were systematically included in each PCR plate. Contamination prevention was rigorously addressed by employing separate workspaces for different stages of the protocol. Additionally, reagents were aliquoted to minimise exposure and reduce contamination risk.

### qRT-PCR analysis for 18S, ACTB, p53, and dicer 1

Quantitative real-time PCR for 18S rRNA, ACTB, p53, and Dicer 1 was performed using specific TaqMan Gene Expression Assays. The reaction setup followed the protocol outlined in Table 2, with a total reaction volume of 5 μL comprising 2.5 μL of TaqMan Universal PCR Master Mix (2X), 0.5 μL of TaqMan Assay for each gene (20X), 1.0 μL of cDNA, and 1.0 μL of nuclease-free water.

The thermal cycling conditions are detailed above in Tables 2 and 5. To ensure assay specificity and integrity, no-template controls and no-reverse transcription controls were included in each assay and PCR plate.

**Table 5:** RT-qPCR cycling conditions

Stage	Cycling conditions	
Denaturation	95 °C, 15 seconds	× 40 cycles
Annealing and elongation	60 °C, 1 minute	
Hold	4 °C	

### Data analysis

Absolute quantitative RT-qPCR data was imported into GraphPad Prism (Version 8.2.1) and Cq values were plotted against sample using column graphs that compare both singleplex and biplex RT-qPCR results. PCR efficiencies were determined using LinRegPCR (v 2021.2) and measured on a scale between 1.0 and 2.0, with 2.0 representing 100% efficiency. PCR efficiencies above 1.5 were determined to be acceptable. A two-sided t-test was used to determine whether a significant difference was observed between singleplex and biplex RT-qPCR reactions (Cq values) and their PCR efficiencies (*p* < 0.05).

## Results

### HPV16 singleplex and biplex RT-qPCR with HPV16-positive cell lines

**Fig. 1:**
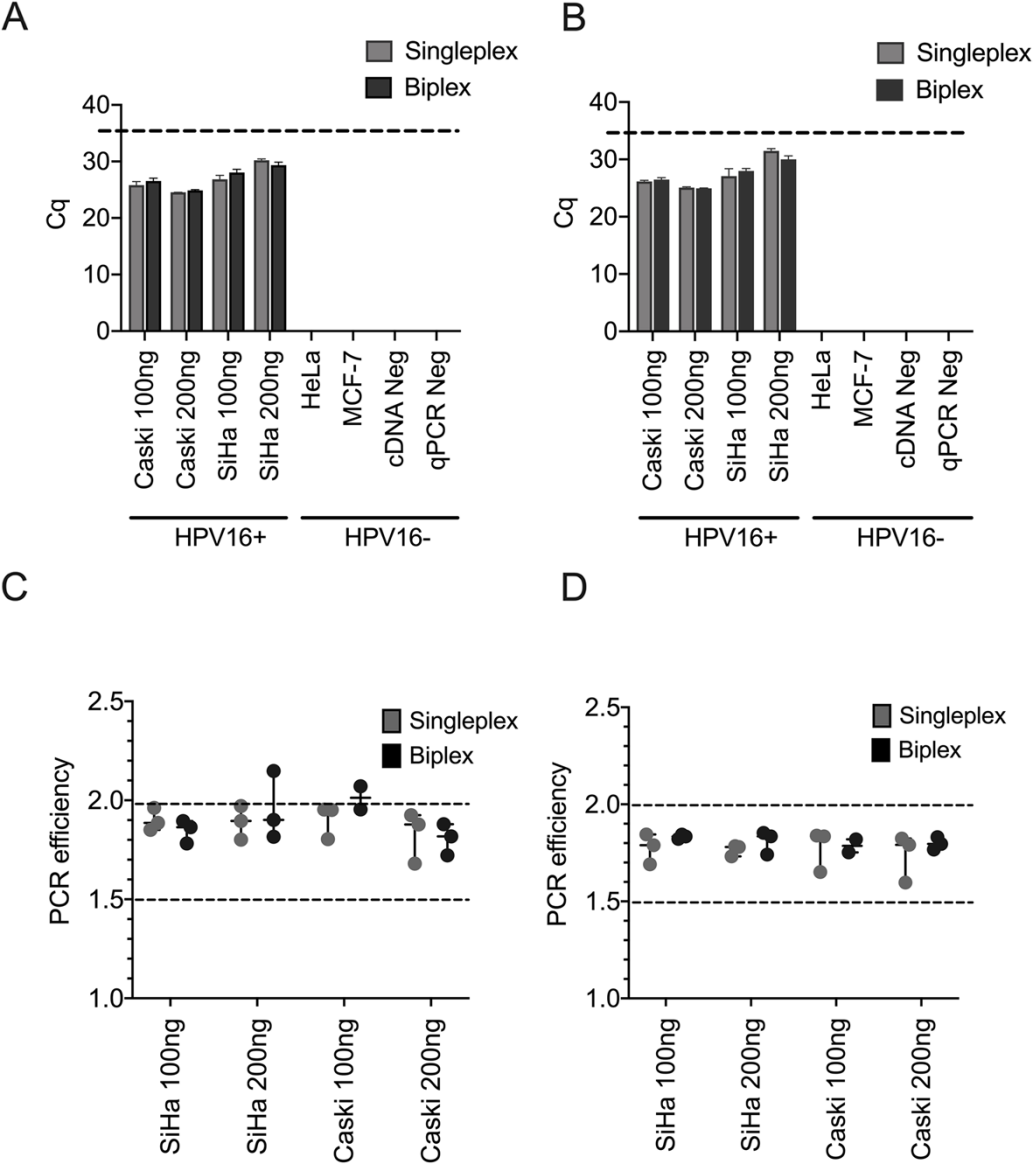
Single and Biplex detection of E6/E7 mRNA in HPV16 positive cell lines. HPV16 positive cell lines included SiHa and Caski, while HPV16 negative cell lines included HeLa (HPV18 positive), and MCF-7 (breast cancer cell line). All reactions were completed in triplicate. **A** E6 plex assay; **B** E7 plex assay. **C** PCR efficiencies for E6. **D** PCR efficiencies for E7 assay. 100% PCR efficiency is depicted at 2.0, with the acceptable threshold of efficiency at 1.5

The E6 and E7 primer/probe combination were first tested by singleplex and biplex RT-qPCR using the HPV16-positive cell lines, SiHa and Caski, and HPV16-negative cell lines, HeLa, and MCF-7. Cq values were measured for two total RNA input amounts, 100 ng and 200 ng. Both E6 and E7 were detected in the HPV16-positive cell lines only (Fig. 1A & B). Despite a 2-fold increase in RNA input, the expression levels of both probes were lower in SiHa cells, which was determined to be due to this cell line only having two viral insertions of HPV16 [35]. Nevertheless, 100 ng of input RNA was found to be adequate to detect both E6 and E7. Furthermore, no significant difference in Cq value was observed between the singleplex and biplex RT-qPCR methods. PCR efficiencies for HPV16-positive results for both the E6 and E7 were calculated using LinRegPCR. All HPV16- positive samples remained above an efficiency of 1.5, and no significant difference was observed between the mean RT-qPCR efficiency of the singleplex and biplex of E6 and E7 mRNA in samples at both 100 ng and 200 ng (*p* value < 0.05) (Fig. 1C & D). Overall, biplexed samples fared better than singleplex in terms of PCR efficiency, particularly for the E7 assay.

### Minimal RNA required for detection of HPV16

**Fig. 2:**
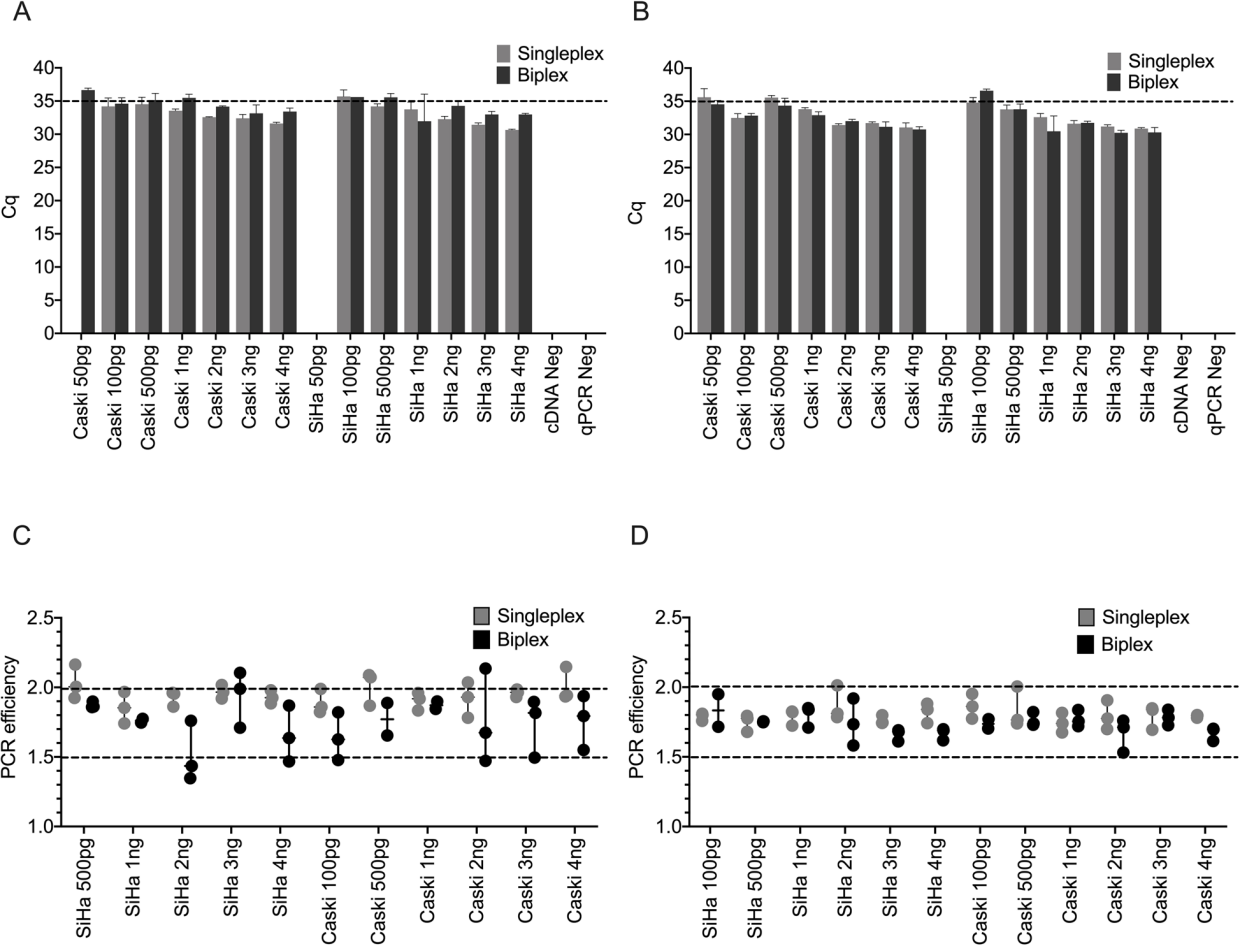
Evaluating total RNA input for E6 and E7 Single and Biplex reactions. **A** E6 plex assay; **B** E7 plex assay. Cq 35 was determined to be a reasonable threshold for a positive result. All reactions were completed in triplicate. PCR efficiencies for **C **E6 assay; **D** E7 at different total RNA inputs. 100% PCR efficiency is depicted at 2.0, with the acceptable threshold of efficiency at 1.5

Next, we investigated the minimal quantity of total RNA required to detect E6 (Fig. 2A) and E7 (Fig. 2B) in SiHa and Caski cell lines. To this end, we prepared a series of RNA dilutions at concentrations of 50 pg, 100 pg, 500 pg, 1 ng, 2 ng, 3 ng, and 4 ng. We established a Cq value of 35 as the threshold for a positive result.

Our data indicated that for both SiHa and Caski, an RNA input of 50 pg in the E6 assay, and for SiHa in the E7 assay, resulted in Cq values exceeding 35, indicating negative results. Notably, upon increasing the RNA input to 100 pg, the Cq values for both SiHa and Caski in the E6 assay, and for SiHa in the E7 assay, were below the 35-cycle threshold, signifying positive results.

Consequently, we concluded that the threshold RNA input for a reliable positive detection in our assays is 100 pg. This represents the lowest RNA concentration that consistently generates Cq values beneath the 35-cycle threshold, thereby indicating a positive result. Notably, no significant disparity in Cq values was observed between the singleplex and biplex methods (*p* < 0.05). We further computed the PCR efficiency for the E6 and E7 assays in HPV-positive samples (Fig. 2C and D). While some variations in the mean RT-qPCR efficiencies were noted, the majority of samples remained above the threshold of 1.5. In samples with minimal RNA input, singleplex reactions exhibited superior PCR efficiencies compared to biplex reactions. It is acknowledged that the analyses to determine the limit of detection (LOD) and the limit of quantitation (LOQ) were not performed. The focus of our investigation was to establish the practicality of detecting HPV16 RNA in saliva samples as an indicator of transcriptional activity.

### Single and biplex detection of E6 and E7 using patient tissue

**Fig. 3:**
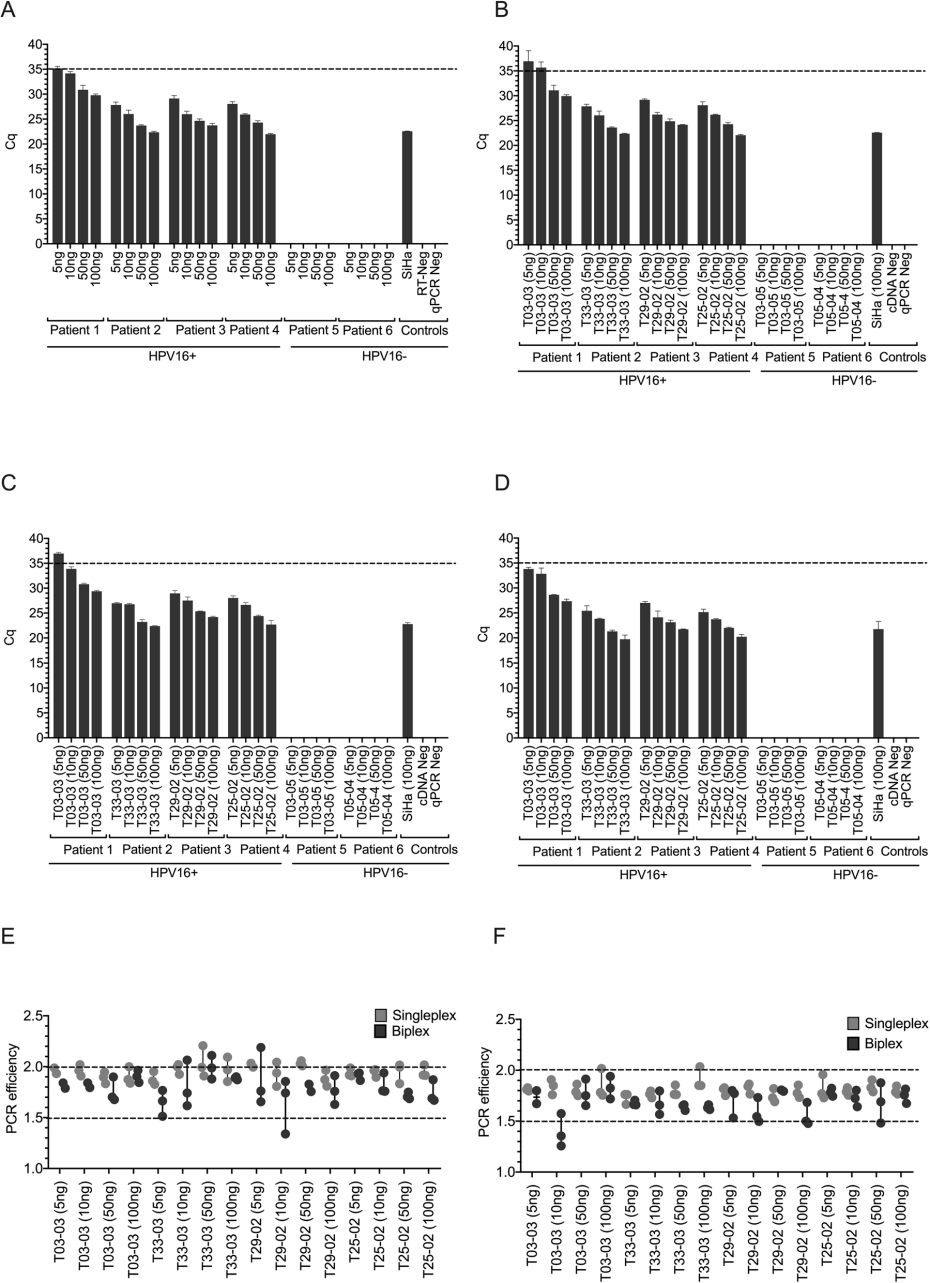
Biplex and singleplex detection of E6 and E7 mRNA in patient tissue. Controls included SiHa cells as a positive control. All reactions were completed in triplicate. **A** Biplex E6 assay; **B** Biplex E7 assay. **C** Singleplex E6 **D** Singleplex E7. **E** PCR effieciencies for E6 and **F** E7 in patient tissue specimens

Subsequently, we evaluated the expression of E6 and E7 in patient tissues, distinguishing between HPV16-positive and HPV16-negative samples (Fig. 3A, B, C & D). The isolated RNA from these samples was serially diluted, resulting in total inputs of 5 ng, 10 ng, 50 ng, and 100 ng for all patient specimens. Both the E6 and E7 assays successfully detected their respective targets in both singleplex and biplex formats. As anticipated, higher amounts of RNA input led to decreased Cq values. In p16 negative patient tissues, no E6 or E7 mRNA was detected. Based on these findings, an RNA input of 10 ng would guarantee a positive result below the designated Cq threshold of 35. Furthermore, PCR efficiencies were determined using LinRegPCR (Fig. 3E & F). While the majority of samples remained above the established threshold of 1.5, we did observed variations in average RT-qPCR efficiencies. In samples with minimal RNA input (i.e., less than 10 ng), singleplex reactions exhibited superior PCR efficiencies compared to biplex reactions. Notably, a sample input of 100 ng of total RNA demonstrated the highest performance, with PCR efficiencies closest to 2.0.

### Detection of viable HPV16 virus in patient saliva

**Fig. 4:**
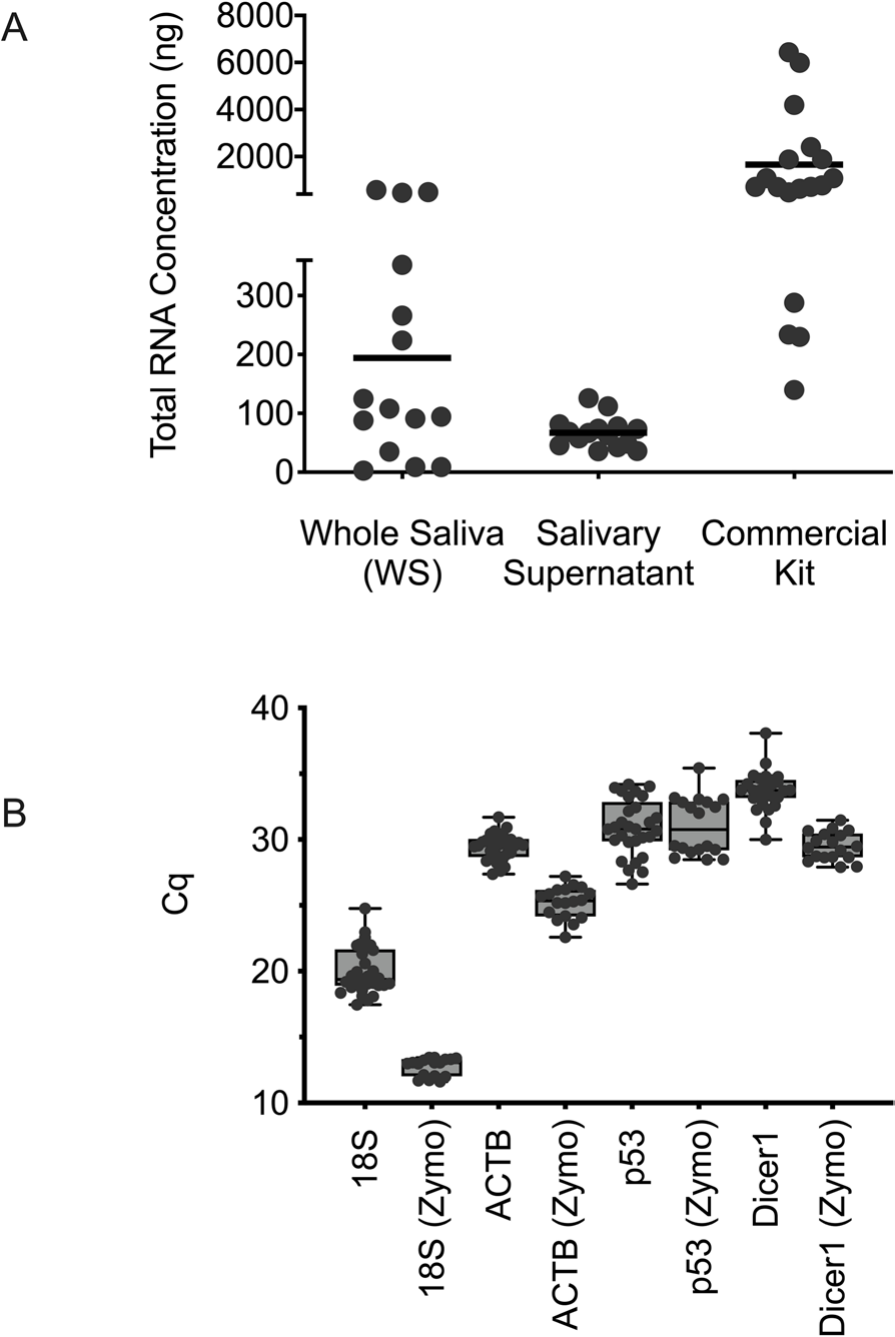
**A** Comparison of total RNA concentration obtained from various saliva collection and processing methods. Whole saliva (*n* = 12) and salivary supernatant (*n* = 12) via the commercial Zymo kit (*n* = 12). **B** Comparison of RNA expression of selected genes in original saliva collection method vs. commercial Zymo tubes. All reactions were completed in triplicate

The collection method for saliva is pivotal for any downstream testing. We compared two standard methods: 1) collecting whole saliva without additives and 2) using a commercial kit with additives. Following collection, we isolated total RNA using our established protocol [36]. Figure 4A shows the RNA concentrations from whole saliva isolations. The average RNA concentration from saliva collected without additives was 67 ng, substantially lower than the 1775 ng average from the commercial kit. Despite the higher yield from the commercial kit, the variation in RNA concentrations was still comparable to that seen in non-additive saliva isolations. It is important to note that these methods were not employed simultaneously on the same samples. RNA was isolated from 28 individuals using the non-additive method and 18 individuals with the commercial kit.

We then determined if the RNA from these commercial kits would provide usable material for PCR amplification. As shown in Fig. 4B, we were able to detect the following targets: 18S, Beta Actin (ACTB), p53 and Dicer1, with RNA collected from both methods. RNA input into the RT-qPCR was standardised to 100 ng. Cq data for the commercial and non-commercial kits were aggregated to understand how the methods compared. For 18S, the mean Cq value for the non-commercial kit was 20.0 and the mean Cq value for the commercial kit was 12.8. Similarly, mean Cq values for ACTB were 29.4 and 25.2 for non-commercial and commercial kits, respectively. Cq values for p53 were similar for both kits, with a mean Cq value of 30.9 for non-commercial and 31.0 for commercial. Mean Cq values for Dicer1 were 33.7 for non-commercial and 29.5 for commercial. Overall, the commercial kit performed better across the various gene targets.

### Biplex RT-qPCR of E6 and E7 mRNA in patient saliva

**Fig. 5:**
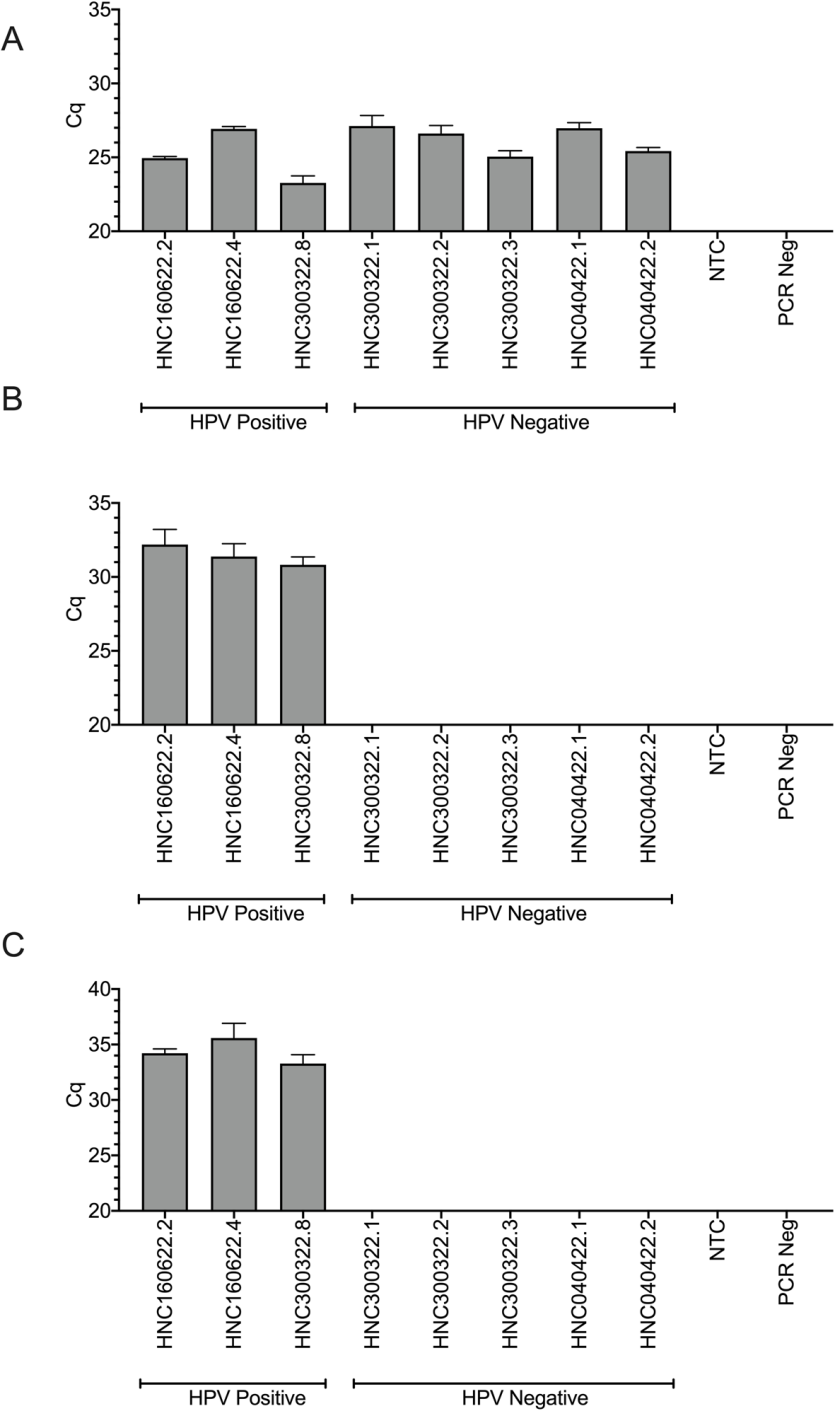
Biplex detection of E6 and E7 mRNA in saliva specimens. RNA was isolated from the saliva of eight HNSCC patients (*n* = 3 p16 positive; *n* = 5 p16 negative). Negative experimental controls included cDNA (NTC) and PCR negative reactions. All reactions were completed in triplicate. **A** Beta 2 Microglobulin (B2M) was used as a positive control and reference gene to ensure expression observed in the E6 and E7 assays was positive and accurate, **B** E6 assay, **C** E7 assay

In the next step, we used RNA isolated from the collected saliva to determine if a biplex RT-qPCR could be used to amplify and detect both E6 and E7 targets (Fig. 5B & C). As a proof of principle, 100 ng of total RNA was isolated from 1.2 mL of saliva from eight HNSCC patient specimens, including three p16 positive and five p16 negative patients. Using a biplex approach, both E6 and E7 were detected in p16 positive samples. The E6 assay (mean Cq 31.5) had slightly increased levels of expression compared to the E7 assay (mean Cq 34.4). As expected, the E6 and E7 targets were not found in the p16-negative patients. Beta 2 Microglobulin (B2M) was used as both a positive reference and was amplified and detected in all samples (Fig. 5A).

**Fig. 6:**
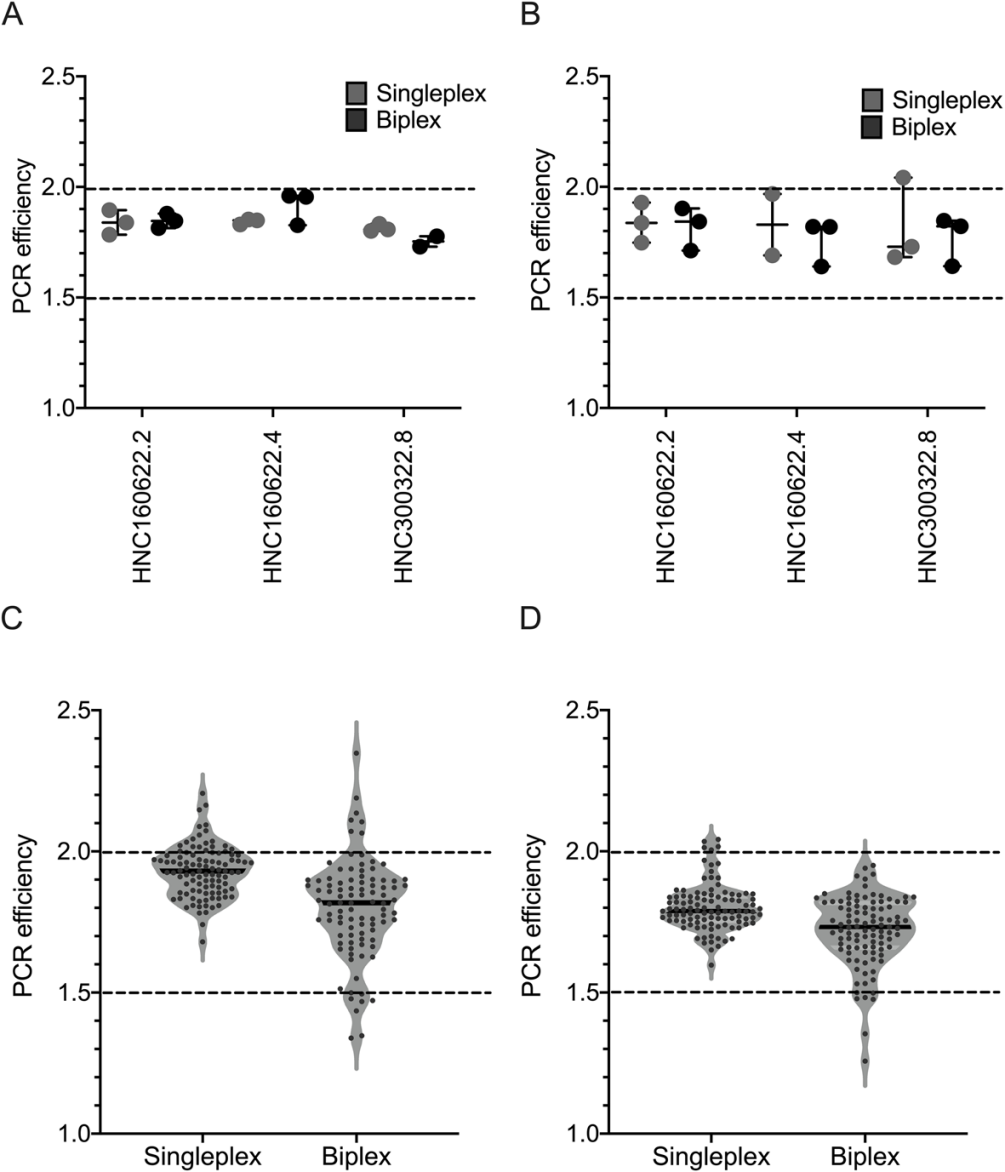
Single and Biplex PCR effieciencies for E6 and E7 in salvary specimens. **A** E6 plex assay; **B** E7 plex assay, 100% PCR efficiency is depicted at 2.0, with the acceptable threshold of efficiency at 1.5. **C** E6 overall PCR effiecinecies and **D** E7 overall PCR efficiencies

PCR efficiencies of the HPV16-positive results for both the E6 and E7 assays were calculated using LinRegPCR (Fig. 6A & B). Overall, both E6 and E7 performed with the same mean PCR efficiency of 1.8 across the two assays and methodologies. The E6 assay showed less variability in PCR efficiency than the E7 assay. All p16 positive patient saliva samples remained above the 1.5 efficiency threshold and there was no significant difference in mean RT-qPCR efficiencies between the singleplexing and biplexing of E6 and E7 mRNA (*p* < 0.05) (Fig. 6C & D).

## Discussion

The improved treatment outcomes and survival rates of HPV-positive OPC patients underline the importance of accurate HPV detection for guiding treatment strategies [37, 38]. While HPV-encoded oncogenes E6 and E7 disrupt cell cycle regulation and are key biomarkers for HPV-associated cancers [39–41], the overexpression of p16, though sensitive, is not entirely specific for high-risk HPV infection [23]. This necessitates a more precise approach, as approximately 5–10% of all OPCs may exhibit false positives due to p16 overexpression unrelated to HPV infection [42–44].

Consequently, the use of p16-IHC or HPV-specific testing alone as a reliable means of determining HPV status has been called into question, with recent studies identifying a subgroup of patients with discordant p16 and HPV positivity [45]. Specifically, most of the discrepant cases reported to date are p16-positive but HPV DNA-PCR or DNA-ISH negative. In light of these findings, the College of American Pathologists (CAP) recommends additional HPV-specific testing at the discretion of the pathologist and/or treating clinician following p16 testing [46]. These developments highlight the need for greater scrutiny of testing methods and the importance of accurate HPV status determination in guiding clinical decision-making.

It is for this reason that viral RNA expression has been suggested as the gold standard for a viable infection, meaning the virus is transcriptionally active. Our study contributes to this need by developing a biplex RT-qPCR method for non-invasively detecting active high-risk HPV16 in saliva. This approach, focusing on E6 and E7 mRNA, offers a potential alternative to p16-IHC and DNA-based HPV tests, enhancing the detection of transcriptionally active infections. Furthermore, this methodology is scalable and well-suited for high-throughput screening, making it an attractive option for widespread screening or HPV16 surveillance programs.

Our results demonstrated successful detection of E6 and E7 mRNA in HPV16-positive cell lines, SiHa and Caski, and in p16-positive OPC patient tissues across a range of RNA input levels. This not only highlights the assay’s sensitivity but also its efficiency in amplifying these specific mRNA targets. The specificity of our assay was further validated in clinical settings: when applied to OPC saliva samples, transcriptionally active E6 and E7 mRNA were exclusively (100%) detected in saliva from p16-positive patients. This specificity is required for the assay’s potential clinical application, ensuring it reliably identifies patients with active HPV16 infections.

Moreover, the assay’s performance in terms of PCR efficiency was robust. We observed mean PCR efficiencies of approximately 1.8, indicative of optimal amplification. This efficiency was consistent across both singleplex and biplex methods for E6 and E7 assays. The slightly better performance of the E6 assay compared to the E7 assay, with mean Cq values of 31.5 and 34.4, respectively, aligns with existing studies suggesting more consistent expression of E6 in various HPV types and stages of infection [47, 48].

Saliva testing offers advantages over blood and tissue-based testing due to its non-invasiveness and ease of sample collection, allowing for a time- and cost-effective diagnosis. Two primary considerations drove the use of tissue biopsies as a comparison in this study. Firstly, tissue biopsies are widely recognised as the ‘gold standard’ in the diagnosis and characterisation of OPCs. By comparing our saliva-based test with tissue samples, we aimed to benchmark our method against the established standard in clinical practice. Secondly, our access to these specific tissue samples, with their well-documented histological profiles, provided an opportunity to validate our saliva test. While brush biopsies are less invasive and easier to collect, they were unavailable for this study. Furthermore, our focus was to compare our saliva-based diagnostic approach with the most rigorous diagnostic method currently available, which, in this case, was tissue biopsy analysis.

Several studies have sought to use saliva for oral cancer detection but very few studies to date have used RNA to detect viable infections [49–56]. In one Australian study, it was demonstrated that saliva rinses could be used to detect key HPV-DNA oncogenic targets with 92.9% sensitivity. It was shown that 39 of 42 oral rinses from p16-positive patients had detectable HPV16-DNA [51]. Another study using oral rinses from 110 patients employed nested PCR to detect low copy numbers and showed a sensitivity rate of 75% [54]. Furthermore, antibodies specific to HPV16 E6 and E7 were present in serum at a similar rate of 51.4%. Although the sensitivity rates were low, it suggests that HPV detection in oral rinses may be comparable with the gold standard method of p16 testing in tumour tissues [53].

The primary limitation of our approach is that RT-qPCR only detects RNA, raising the potential for false negatives, particularly if the virus is dormant and not transcriptionally active. To enhance diagnostic accuracy, this salivary RT-qPCR could be used alongside p16 staining. A dual positive result from both tests might provide more clinical insight that relying on p16 staining alone. Future enhancements of this assay could include additional HPV16 targets to address these limitations.

One possible approach is to identify HPV16 genes associated with viral dormancy and include these targets along with E6 and E7. The E2 gene is frequently overexpressed during viral latency and a key regulator of both E6 and E7 [57]. The triumvirate of E2, E6, and E7 targets might be able to discern between viable and latent viral infection. Another strategy is to detect both the presence of viral DNA and RNA in the same RT-qPCR assay. Other viral DNA targets could include the L1 and L2 genes which are highly conserved [58, 59] or non-transcribe regions of the HPV16 genome. The latter would be an ideal RT-qPCR target.

In this study, we focused on demonstrating the feasibility of detecting HPV16 RNA in saliva, a significant step toward non-invasive diagnostics. We acknowledge that the determination of limit of detection (LOD) and limit of quantitation (LOQ) was not performed, a decision shaped by our aim to primarily assess qualitative detection capabilities. While our results highlight the assay’s potential sensitivity, the absence of LOD and LOQ analyses represents a limitation. Future research should address this by quantifying the assay’s sensitivity and expanding its clinical applicability, thereby expanding  our understanding of HPV16’s detection dynamics in saliva and its implications for early diagnosis and monitoring.

We also acknowledge that the sample size used for the salivary testing is limited and a larger cohort will be required to further assess the sensitivity and specificity of this salivary RT-qPCR method. An additional hurdle in utilising salivary samples is the absence of universally recognised standards for the collection and handling of such specimens. A consistent collection protocol and a reliable approach for extracting genomic material must be established to address this issue [36]. Presently, only a limited number of techniques are available that can extract both DNA and RNA from a single salivary sample [60, 61].

Overall, continued efforts towards standardisation and optimisation of saliva-based testing will be important for advancing the field of liquid biopsy and improving patient diagnosis. Despite these challenges, the use of saliva in HPV16 testing continues to show promise. Ongoing efforts to standardise salivary collection, processing, and inclusion of other RNA/DNA targets, will be critical in developing a robust RT-qPCR liquid assay for HPV detection.

## Data Availability

Data is provided upon request. Please contact Nham Tran, email: nham.tran@uts.edu.au.
